# Enhanced Short-Term Memory Function in Older Adults with Dementia Following Music-Feedback Physical Training: A Pilot Study

**DOI:** 10.3390/brainsci12091260

**Published:** 2022-09-16

**Authors:** Jessica V. Strong, Maria Arnold, Lydia Schneider, Johanna Perschl, Arno Villringer, Thomas Hans Fritz

**Affiliations:** 1 Department of Psychology, University of Prince Edward Island, Charlottetown, PE C1A 4P3, Canada; 2 Max Planck Institute for Human Cognitive and Brain Sciences, Stephanstrasse 1A, 04103 Leipzig, Germany; 3 Institute for Psychoacoustics and Electronic Music (IPEM), Blandijnberg 2, 9000 Ghent, Belgium

**Keywords:** music therapy, long-term care, dementia, physical activity, older adults

## Abstract

Prior research demonstrates that music making, physical exercise, and social activity have unique, positive effects on cognition and mood. One intervention, “Jymmin^®^”, was developed incorporating these approaches and found effective for decreased pain perception and increased endurance, self-efficacy, mood, and muscle efficiency. Previously, Jymmin was not piloted with older adults with dementia. The current study is a randomized pilot study of the Jymmin^®^ with an older adult population in a long-term care facility (*n* = 38), evaluated across dementia levels (mild, moderate, or severe). Results found significant improvements in scores on a confrontation naming task across all conditions (*p* = 0.047) and a significant interaction effect for short-term memory scores (*p* = 0.046), suggesting higher scores at Time 2 for the experimental group and at Time 3 for the control group. There were no significant changes in mood ratings. Findings are discussed in the context of neural activity and musical agency.

## 1. Introduction

Demographic trends show the global population of adults over the age of 60 is projected to double from 1 billion in 2020 to 2.1 billion in 2050, and older adults already outnumber children under the age of 5 [1,2]. Age is the most significant risk factor for developing dementia [2], and beginning around the age of 80, prevalence rates of dementia rise rapidly [3]. The World Health Organization recognizes dementia as “one of the major causes of disability… among older people worldwide,” noting an impact on functioning across a variety of domains (e.g., physical functioning, social functioning, etc.). Approximately 55 million people in the world have dementia [2]. In the United States, 40–50% of the 1.4 million individuals living in long-term care facilities have been diagnosed with dementia [4].

Individuals with dementia residing in long-term care facilities are often prescribed medications for cognitive impairment (e.g., cholinesterase inhibitors and glutamate antagonists) and/or behavioral symptoms of dementia (e.g., neuroleptics or antidepressants). Indeed, between 20% and 50% of long-term care residents battling dementia receive at least one prescribed psychotropic medication [5,6,7]. However, not a single pharmaceutical treatment has successfully altered the course of Alzheimer’s disease [8]. Recognizing the shortcomings of medication in curing cognitive decline, there has been a renewed focus on non-pharmaceutical interventions (e.g., lifestyle, environmental, or acupuncture [9]). Research has supported a variety of factors that may boost cognitive functioning in older adults, including social activity [10], physical activities, aerobic exercise [11,12,13], and music making [14,15]. Each of these has independently shown a benefit in delaying the onset of diagnosis, reducing the risk of developing cognitive impairment or slowing cognitive decline in already diagnosed individuals.

### Cognitive Functioning in Long-Term Care Facilities and the Current Study

Research has shown that physical activity, as well as social activity, may protect against cognitive decline in late life [11,16,17]. Physical fitness is positively related to learning and memory, increased processing speed, and responsiveness mainly due to intensified signal transduction between neurons [18,19]. There is some evidence that an active lifestyle, including physical activities, promotes healthy cognitive functioning in at-risk older adults and can lead to positive long-term effects [12,20]. The most striking findings on the relationship between social activity and cognition have recently examined the relationship between social isolation and a host of negative health effects. For example, studies find that lack of social activity can impair day-to-day functioning, reduce physical activity levels, impair cognitive performance, affect the progression of dementia, or increase the likelihood of long-term care admission [21,22]. Adults who are engaged in more social activities are at a reduced risk of developing dementia [23].

In the case of music engagement and music therapy, research has found that music evokes both emotional and physiological reactions [16]. In fact, interest and demand for actively making music have doubled since 2000 among the over 60s cohort [24]. Music is closely linked with both emotions and memory [25], providing promising effects for using music to increase positive emotions and spark memories in older adults with dementia, and decreasing behavioral symptoms of dementia [26]. Neuropsychological test results of 89 dementia patients (with mild and moderate dementia) revealed enhanced mood and quality of life for those treated using music interventions [27]. There have been a few approaches that combine existing interventions for physical, social, and music activities. MAKS-therapy, for instance, is a concept developed specifically for the needs of older adults with dementia, consisting of motor skills (M), everyday practice of activities (A), cognition (K), and spirituality (S; e.g., singing church songs). The current intervention (Jymmin) was also designed to integrate active music-making with physical exercise in small groups of two to three individuals. Studies with younger and middle-aged adults have found significant effects after participating in Jymmin on pain perception [28], muscle efficiency, and perceived exertion during exercise [29], increased mood [30], an increase in self-efficacy in poly substance users [31] and increased divergent thinking capacity [32].

The goal of the current study was to pilot an intervention incorporating each of these three elements (i.e., aerobic exercise, social activity, and music-making; “Jymmin”) modified for older adults with cognitive impairments residing in long-term care facilities. Although Jymmin has been tested with healthy younger and middle-aged adults, it has not yet been tested with older adults with cognitive decline. Our pilot study tested the impact of Jymmin and physical exercise while listening to music. We hypothesized that (1) there will be an impact on mood and cognitive functioning of participation across time (in both conditions), (2) individuals will experience an effect on mood and cognition following participation in the experimental condition, and to a lesser degree following the control condition, and (3) there will be an interaction effect of dementia (mild, moderate, severe), and group (control vs. experimental) on mood and cognitive outcomes.

## 2. Methods

### 2.1. Participants

Residents of local urban long-term care facilities (*n* = 38 total, 30 female) in a large city in Germany participated in the current study, all German-speaking. Ten participants were excluded due to absences (*n* = 9) or declining to participate in the mood or cognitive assessment (*n* = 1). One resident was excluded for demonstrating normal cognition. Participants were not excluded for any physical limitations (e.g., hearing impairment, paresis) as modifications were able to be made to the intervention as needed based on individual abilities.

A total of 27 participants were included in the data analyses. Participants were older adults (*M* age = 81.97, *SD* = 7.04; range 63–92) who underwent cognitive screening for dementia staging, based on Mini-Mental State Examination (MMSE; Folstein, Folstein, and McHugh, 1975) scores. Dementia staging was classified as mild (*n* = 13, MMSE = >18), moderate (*n* = 16, MMSE = 10–17), or severe (*n* = 8, MMSE < 10), based on the DEGAM classification system [33]. No specific etiologies of dementia were determined as it was not available for all participants as part of their medical history and was outside the scope of our cognitive testing.

### 2.2. Materials

The Multidimensional Mood Questionnaire [34] is an instrument used for self- or third-party assessment of current mood. The short version A that was used in the current study includes 13 items (3 subscales, comprised of 4 items and a total score), with each item listing a mood word (e.g., “satisfied”). Each item is scored on a 5-point Likert scale ranging from 1 (“not at all”) to 5 (“very”). The three subscales include “Good-Bad Mood,” “Alertness-Tiredness,” and “Calmness-Agitation.” Total scores ranged from 4 (poor mental health condition) to 20 (good mental health condition). The MDMQ has been found to be highly reliable (Cronbach’s alpha = 0.73–0.89 [35]).

The Short Test Capturing Cognitive Performances (KKL; Kurztest zur Erfassung kognitiver Leistungen [36]) has three versions for repeated measures (A, B, and C), including several tasks and tests to measure cognition across a few domains, including selective attention, processing speed, memory (short- and long-term delay), confrontation naming, and motor coordination.

### 2.3. Procedures

Participants were enrolled in the study in accordance with the regulations established by the Helsinki Declaration of the World Medical Association [37]. Specifically, given that participants with dementia were regarded as incapable of giving informed consent, informed consent from the legally authorized representative was sought in writing. Representatives were adequately informed of the aims, methods, sources of funding, any possible conflicts of interest, institutional affiliations of the researchers, the anticipated benefits and potential risks of the study and the discomfort it may entail, and any other relevant aspects of the study. They were informed of the right to refuse to have their relatives participate in the study or to withdraw consent to participate at any time without reprisal. Participants with dementia were included in this research study due to the high likelihood of benefit for them (based on prior research), both by motivating them during health-promoting physical exercise and resulting in positive psychological effects regarding aspects of the functionality of memory. While consent of the legally authorized representative was acquired, it was a priority that a participant’s dissent to engage during the intervention (e.g., when becoming bored) was to be respected. The research study could not instead be performed with persons capable of providing informed consent because the mental condition that prevents giving informed consent is a common characteristic of the research group. The research entailed only minimal risk and minimal burden. Data were anonymized such that they could no longer be associated with an individual in any manner.

**Figure 1:**
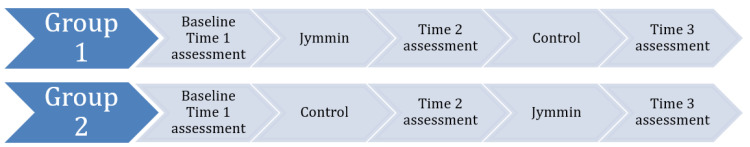
Cross-over experimental design.

We randomly assigned older adults with different levels of cognitive impairment (mild, moderate, severe dementia) to an active experimental group (Jymmin) or to a control group doing physical exercise while passively listening to music. Residents completed an interview to collect general demographic information, the MDMQ, and the KKL. In this cross-over pilot study, participants were randomly assigned to receive 6 min of either the intervention or the control (exercise with passive music listening) across 5 consecutive days. After one week of the assigned first condition, the participants “crossed-over,” completing a second week in the other condition (See Figure 1). Training sessions were completed with two individuals who were in the same treatment condition and began with the experimenter putting an exercise band (i.e., a Thera-band) in the participants’ hands, providing the instructions, “Please sit down facing your training partner. Pull the Thera-band in a way that is comfortable to you.” Every two minutes, the experimenter prompted the participants “Good. Please continue pulling the Thera-bands.” After completing 5 days in each condition, current mood and cognitive state of each participant were measured a second and third time. In total, participants completed a baseline assessment of mood and cognitive state, a second evaluation between conditions, and a final evaluation.

### 2.4. Intervention

**Figure 2:**
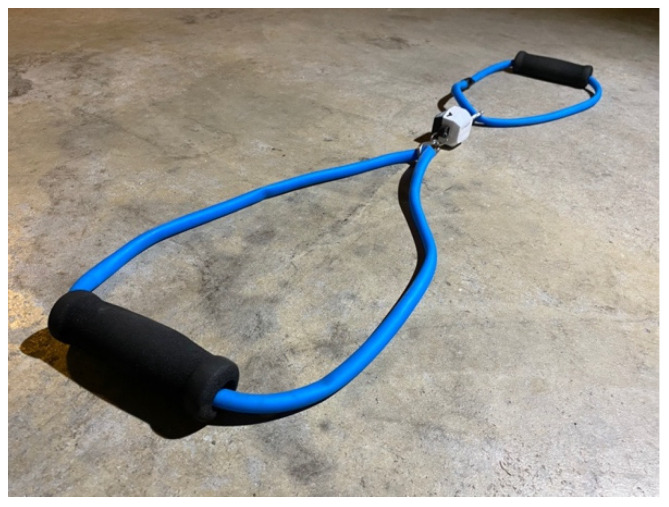
Thera-band.

The Jymmin intervention was developed by the corresponding author (TF) and tested with younger adults [28,29,30,31,32] before modification for older adults primarily by using Thera-bands rather than traditional exercise equipment (Figure 2). Thera-bands were tied to the ceiling or bed and were fitted with sensors, which mapped exercise movements to a musical feedback software (Jymmin GmbH, Leipzig, Germany; www.jymmin.com; accessed on 1 June 2018). Otherwise, the software for the intervention was the same.

The sensor can report movement to a computer, tablet, or smart phone (here, a tablet was used) system that translates the sensor output into a music signal. The music can either change continuously, for example, varying a cutoff filter, or use thresholds (this means that participants must reach a certain threshold of force or work before the music signal changes) to translate the movement data into an auditory signal or both. In the setup, three components were used (1) two sensors connected to two rubber bands played by two people, (2) a movement-analysis and music-processing device, and (3) a speaker. The musical output was created from a set of pre-made musical “loops”, which can be up to several minutes long. Using patented software, the performers could, through their physical engagement, navigate between such musical loops, for example blending in new loops with increased energy expenditure or multi-crossfading between several loops along a dimension of exertion. Using a composition tool developed by the Jymmin GmbH this dimension of exertion was coupled to multi-player music “instruments” that could be played quite intuitively, ranging from more relaxed versions of a musical loop to more energetic versions of the same or similar loop. This control of the musical output is thus achieved through a combination of setting thresholds for when the musical “loop” changed from one to another and thresholds determining the on and offset of an acoustic filter which gradually changes the music. The musical compositions were composed so that the music performed by the two players always fit together with respect to tonality and tempo. We changed the style of music across sessions, including a disco samba style influenced by pop music, classical instruments, or mixed guitar and trumpet.

Participants in the control condition listened to similarly styled music, pulling at Thera-bands that were not connected to the musical composition software. This condition was the control and similarly consisted of physical exercise in groups of two while passively listening to music. In order to ensure that all participants could hear the sound, a speaker system was used.

### 2.5. Data Analysis

Analyses were run on SPSS Version 25. MDMQ subscales were averaged and scored according to the manual. KKL scores were normed for age. Assumptions for variables were checked prior to running analyses. We ran repeated-measures analysis of variance (ANOVA) to test the impact of training for all participants across time (Hypothesis 1). We ran a paired *t*-test to analyze the effects of the intervention (Jymmin’ vs. passive control) on cognitive test scores and mood ratings (Hypothesis 2). Finally, we used Factorial ANOVA and non-parametric Kruskal–Wallis test for non-normally distributed data to analyze the impact of Cognition (mild, moderate, severe stage dementia) and Treatment Group (intervention vs. control) on cognitive test scores and mood ratings (Hypothesis 3). We used an alpha cut-off value of 0.05, while also considering effect sizes, e.g., partial η^2^, in our interpretation of the data given the sample size in this pilot study.

## 3. Results

As expected, there were significant differences on cognitive scores across dementia groups (i.e., mild, moderate, severe) on Selective Attention I (Numbers: *F* (2, 24) = 9.79, *p* = 0.001; Letters: *F* (2, 24) = 11.60, *p* < 0.001), Short-Term (*F* (2, 24) = 4.92, *p* = 0.02) and Long-Term (*F* (2, 24) = 3.63, *p* = 0.04) memory (STM, LTM). We did not find significant differences among groups on tests of Confrontation Naming (*F* (2, 24) = 1.03, *p* = 0.37), Selective Attention II (*F* (2, 24) = 2.80, *p* = 0.08), or Coordination (*F* (2, 24) = 3.27, *p* = 0.056). In addition, on the MDMQ, there were no significant differences on any subscale across stages of dementia (Good-Bad: *F* (2, 19) = 1.56, *p* = 0.24; Alert-Tired: *F* (2, 18) = 0.82, *p* = 0.46; Calm-Agitated: *F* (2, 19) = 0.37, *p* = 0.69). See Table 1 for baseline scores per cognitive group.

### 3.1. Change over Time

See Table 2 for changes in scores over time. A repeated-measures ANOVA across three time points, with two-level between subjects factor of Treatment Group (i.e., experimental or control) revealed that participants showed no significant change on the MDMQ scores over time on any subscale (Good-Bad: *F* (2, 42) = 1.12, *p* > 0.05, partial η^2^ = 0.05; Alert-Tired: *F* (2, 40) = 0.30, *p* > 0.05, partial η^2^ = 0.02; Calm-Agitated with Huynh-Feldt correction (Mauchley’s W = 0.676, *p* = 0.03) = *F* (1.61, 30.65) = 0.29, *p* > 0.05, partial η^2^ = 0.02).

Repeated-measures ANOVAs across three time points with between subjects factor of Treatment Group revealed no effect on Selective Attention I (Numbers: *F* (2, 46) = 0.59, *p* = 0.56, η^2^ = 0.03; Letters: *F* (2, 46) = 3.02, *p* = 0.058, partial η^2^ = 0.12), Selective Attention II (*F* (2, 42) = 0.39, *p* = 0.67, partial η^2^ = 0.02), LTM (LTM; *F* (2, 44) = 1.33, *p* = 0.27, partial η^2^ = 0.06) or Coordination (*F* (2, 46) = 0.94, *p* = 0.40, partial η^2^ = 0.04).

**Figure 3:**
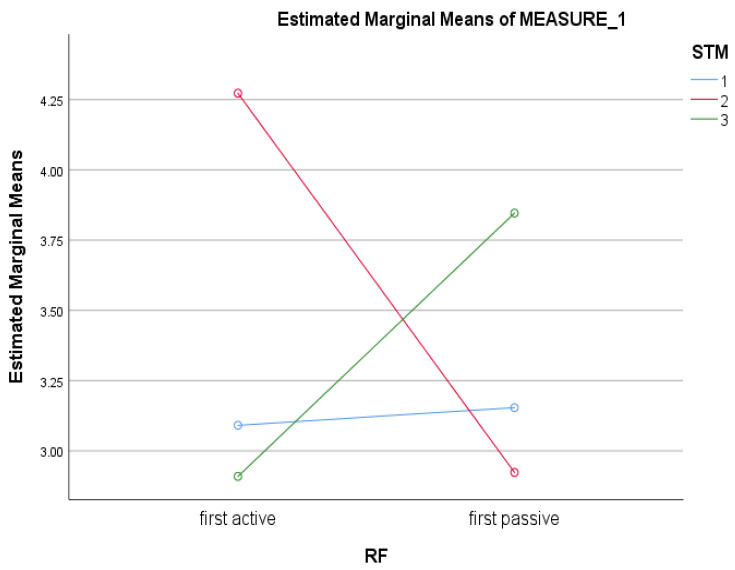
Interaction effect of Time × Treatment Group on Short term memory scores.

There were significant differences on a test of Confrontation Naming across time (correct items: *F* (2, 44) = 3.29, *p* = 0.047, partial η^2^ = 0.13). Post-hoc tests with Bonferroni corrections revealed that scores significantly improved from Time 1 to Time 2 (Mean Difference: −0.89, *p* = 0.049). However, there were no significant differences between Time 2 and Time 3 (Mean Difference = 0.86, *p* = 0.10). In addition, there was a significant interaction term for STM across Time × Treatment Group (*F* (2, 44) = 3.32, *p* = 0.046, partial η^2^ = 0.13). Examination of profile plots showed that while scores on STM at Time 1 were consistent across conditions (i.e., active or passive first), scores on STM at Time 2 were higher for the active first group and at Time 3 were higher for the passive first group (see Figure 3).

### 3.2. Effect of Intervention

Paired samples *t*-tests (see Table 3) showed no significant differences in responses of active compared to passive conditions on MDMQ for Good-Bad (Mean Diff = −0.20, *SE* = 0.17, *t*(22) = −1.15, *p* = 0.26), Alert-Tired (Mean Diff = 0.15, *SE* = 0.25, *t*(21) = 0.58, *p* = 0.57) or Calm-Agitated (Mean Diff = −0.15, *SE* = 0.30, *t*(21) = −0.49, *p* = 0.63).

On tests of cognition, there were no significant effects on Selective Attention I (correct numbers: Mean Diff = 0.17, *SE* = 0.53, *t*(23) = 0.31, *p* = 0.76; correct letters: Mean Diff = −0.25, *SE* = 0.69, *t*(23) = −0.36, *p* = 0.72), Selective Attention II (Mean Diff = 8.48, *SE* = 7.01, *t*(23) = 1.21, *p* = 0.24), Confrontation Naming (Mean Diff = 0.71, *SE* = 0.41, *t*(23) = 1.73, *p* = 0.10), LTM (Mean Diff = 0.08, SE = 0.44, *t*(23) = 0.19, *p* = 0.85), or Coordination (Mean Diff = 0.04, *SE* = 0.19, *t*(23) = 0.21, *p* = 0.83). There were significant differences on a test of STM (Mean Diff = 1.13, *SE* = 0.48, *t*(23) = 2.33, *p* = 0.03), such that mean scores after a week of the Jymmin’ intervention were significantly higher (*M* = 4.04) than mean scores after a week of the passive listening condition (*M* = 2.92).

### 3.3. Dementia × Treatment Group Effects

We used a 2 × 3 Factorial ANOVA (Treatment Group–active or passive condition first × Cognitive Status) to examine the intersection of these factors on mood ratings and cognitive scores. There was no significant interaction of Cognitive Status × Treatment Group for any mood subscales, including Good-Bad (*F* (2, 16) = 1.64, *p* = 0.23, partial η^2^ = 0.17), Alert-Tired (*F* (2, 15) = 0.94, *p* = 0.41, partial η^2^ = 0.11) or Calm-Agitated (*F* (2, 14) = 0.08, *p* = 0.92, partial η^2^ = 0.01). There were no significant interactions for tests of Cognitive Status × Treatment Group on tests of Selective Attention I Numbers (*F* (2, 18) = 0.83, *p* = 0.83, partial η^2^ = 0.02), Confrontation Naming (*F* (2, 18) = 0.10, *p* = 0.91, partial η^2^ = 0.01), STM (*F* (2, 18) = 0.09, *p* = 0.92, partial η^2^ = 0.01), LTM (*F* (2, 18) = 0.02, *p* = 0.98, partial η^2^ = 0.002), Selective Attention II (*F* (2, 18) = 0.03, *p* = 0.97, partial η^2^ = 0.003), or Coordination *F* (2, 18) = 0.11, *p* = 0.90, partial η^2^ = 0.01).

Selective Attention I Letters violated assumptions of equality of variances, so non-parametric Kruskal–Wallis tests were used. Results from the Kruskal–Wallis analysis recommended rejecting the null hypotheses for scores on Selective Attention I Letters across Treatment Groups (active first *p* = 0.03; passive first *p* = 0.003). In other words, Kruskal–Wallis indicated that for Selective Attention I Letters, there were significant differences in scores between intervention and control conditions.

## 4. Discussion

The aging population is growing, and with it, the need increases to develop non-pharmaceutical interventions that are able to reduce the incidence and support the treatment of cognitive impairments in old age. The current study, therefore, piloted an examination of the effect of a recently developed musical feedback training (Jymmin^®^) on the cognition of participants across dementia stages. The primary findings of the study indicate that (1) participants experienced a positive effect on language (i.e., confrontation naming), regardless of condition, (2) an effect of the intervention on short-term memory, including interaction of group × cognition, and (3) no effect on mood across conditions. Specifically, the interaction found an effect of higher STM scores that was detected following the active condition. Although were no differences at Time 1 on STM scores, people who were in the active condition first showed high scores on Time 2 (immediately following active intervention), whereas people in the active condition second showed high scores on Time 3 (immediately following the active intervention). The effect did not last for people who then began the passive condition second, their Time 3 scores were low again. Our findings related to cognition are consistent with prior studies of physical activity, social activity, and music intervention. Previous research has found that adults who are physically active, or increase their activity, maintain higher cognition for longer compared to less active peers [38]. Social activity, or lack of it, has long been supported to decrease cognitive functioning and increase the risk of dementia [21,22,23]. Finally, Bugos (14,15) has found support across studies for the impact of music making on cognitive functions. Our null findings for mood across conditions are not consistent with the previous literature. In fact, Särkämö and colleagues [27] found that actively making music (e.g., singing) had a significant effect on effect on mood and quality of life. There are a variety of mechanisms and limitations to be considered in contextualizing our findings, including neural activity or musical agency. Additionally, Jymmin incorporates physical and social activity with music making, and as such, each of these components or their combined effects may be implied as mechanisms.

### 4.1. Potential Mechanisms

Although neuroplasticity is proposed as an underlying mechanism for cognitive effects of music-associated training [39,40], it was suggested that enhanced cognitive capacities due to neuroplasticity over the course of an intervention would be more common in younger adults. Cognitive improvements following interventions in older adults may more strongly relate to changes in neural activity in accordance with a shift of strategies not necessarily relying on extensive neuroplasticity. In our pilot data, a shift in neural activity by participating in the active intervention could help explain the short-term memory findings that did not last after discontinuing the active intervention. A training intervention that could induce changes in neural activity beneficial to cognitive capacities would be of great clinical value, especially in the aging population, aiming ultimately at improving cognitive and functional limitations due to neurodegenerative diseases.

Further attempting to explain the short-term effects observed in this study, the effect of musical agency (control over sound) should be taken into consideration [28]. Musical agency is described as “…a sense that [one] can initiate and carry out their own musical ideas…” [41] (p. 103). This concept has been studied throughout the music education literature as children incorporate music into their play from an early age [41]. The concept of musical agency relates to the fact that making music, coupled with physical movement, is observed in traditional celebrations across cultures and is related to greater physical endurance and euphoria (“flow”/“runner’s high”) [42]. Part of the effectiveness of musical agency may also lie in the social aspects included in these gatherings, be it a traditional celebration or Jymmin training.

The curative properties of social activity and societal inclusion of older adults should not be underestimated and could play an important role, not only in the rehabilitation generally but also in the underlying mechanisms of Jymmin. In contrast to social fulfillment, loneliness has received increasing attention for its role in well-being [43] and cognition in late life [44]. Jymmin combines behaviors (social, physical, music making) that have been shown throughout the literature to have a beneficial impact on cognition and mood in older adults. Specifically, Jymmin has participants creating music together while pulling Thera-bands in groups of three individuals. Social interactions naturally arose from sharing a space together and completing a shared musical activity. Older adults show higher life satisfaction when their social behaviors are consistent with earlier life social preferences [45]. Jymmin provided an easy way for older adults in long-term care facilities to connect socially and choose their social role in the group. In a musical interaction, it may be easier for an individual to identify with a social role that fits their earlier life preferences. For example, in the Jymmin group, some individuals could choose to be “musically talkative”, or alternatively, enjoy the company more quietly while musically supporting another in the group. A related concept, “floating intentionality” [46], allows for individuals to identify as a group with a shared musical experience and values while interpreting the experience through their own individual or cultural lens.

In many cases, it may be difficult for older adults to maintain strong social connections through purposeful or meaningful activities in long-term care. Meaningful social activities provide individuals with a stronger sense of accomplishment [10] and opportunities for these achievements to be recognized [47]. Social activities alone may benefit physical health through body stimulation, encouraging an increased range of motion and muscle tone [10]. When social activities are experienced with the aim to create something beautiful and aesthetically pleasing to all participants, a sense of accomplishment and motivation to engage in a physically challenging task are probably especially high.

The literature on the impact of physical activity alone on cognition has mixed findings. Whereas many studies find a benefit to cognition for participation in aerobic activities like walking or biking [17,20,38], other studies fail to find such support [48]. There are a variety of considerations for null findings, including failure to report the specific type of dementia included in the study, the dose and intensity of the physical activity plan, and additional barriers to participating in physical activity for long-term care residents. In the current study, the Jymmin intervention allowed residents to control the intensity of their workout. They controlled the speed at which they worked out, and Thera-bands allowed them to also control the difficulty. Whereas the findings related to the impact of physical activity alone on cognition have been encouraging but mixed, our pilot study suggests that the combination of physical activity with social activity and musical agency may have a positive impact on some aspects of cognition, like attention or short-term memory.

### 4.2. Limitations and Future Directions

Results are preliminary but important given that this was a pilot study and the first to use Jymmin with this population and in the long-term care setting. There are several important considerations. First, the study had low power stemming from a relatively small sample size. Although close to 40 individuals were recruited, 10 had to be excluded due to problems with data or data collection. Research with a vulnerable population of institutionalized older adults with dementia will necessarily have high rates of attrition due to personal factors like illness, fatigue, or acute medical co-morbidities (e.g., delirium), as well as systems or facility level barriers like competing appointments with doctors or family visits [49]. Despite the small sample size, we found significant differences in scores on a short-term memory task and statistical trends (*p* < 0.10) with medium effect sizes in the domains of selective attention and language. The medium effect sizes indicate that although the study may have been underpowered due to a small sample and therefore did not reach statistical significance, there is an effect of medium importance in the cognitive areas of selective attention and language that may be significant in larger, more heterogeneous samples.

Second, mood was measured differently than in previous studies, where it was measured on the same day as training or pre- and post-intervention. Therefore, we were not able to compare alterations in mood due to the intervention with the current design. Mood was measured at three time points, one week apart, and change in mood was most likely to occur directly following the training. Future studies with Jymmin in older adults may take a brief affect or mood assessment directly prior to and after completing each training session.

## 5. Conclusions

Taken together, the positive results of the current study found that after participating in Jymmin, residents’ performance on short-term memory and attention measures improved. The current findings from our pilot study indicate Jymmin is a training that is feasible with older adults with dementia residing in long-term care, and a benefit of participating in this type of active music training can be seen in multiple cognitive domains. Further research will be needed to better understand these cognitive changes and take stronger methodological approaches to detect and understand changes in mood in this specific population from the same training.

## Figures and Tables

**Table 1:** Baseline scores per group based on cognitive status.

Domain	Mild (n = 10)	Moderate (n = 11)	Severe (n = 6)
MDMQ			
* Good-Bad	4.00 (0.91)	4.39 (0.47)	2.94 (1.01)
Alert-Tired	3.92 (0.93)	3.61 (1.17)	2.69 (1.25)
Calm-Agitated	3.78 (1.14)	3.84 (1.16)	4.33 (1.15)
KKL			
** Selective Attention I: Numbers	6.40 (3.13)	5.55 (3.11)	0.33 (0.52)
** Selective Attention I: Letters	7.10 (2.51)	4.45 (3.59)	0.17 (0.41)
Selective Attention II	78.18 (41.59)	84.30 (31.25)	40.91 (50.55)
Confrontation Naming	6.00 (3.23)	4.73 (2.24)	4.17 (2.40)
* Short-term Memory	4.20 (2.53)	4.10 (2.70)	0.67 (1.21)
* Long-Term Memory	2.70 (2.36)	1.55 (2.02)	0.00 (0.00)
Coordination	2.80 (0.43)	2.82 (0.40)	2.00 (1.26)
Length of Testing	12.70 (6.09)	14.18 (4.24)	12.17 (2.48)

Note: * Significant differences based on *p* < 0.05. ** Significant differences based on *p* < 0.01. Length of testing is presented in minutes. Selective Attention II task is in seconds. All other scores are correct responses.

**Table 2:** Scores across three time points.

Domain	T1	T2	T3
MDMQ			
Good-Bad	4.01 (0.93)	4.11 (0.76)	4.30 (0.80)
Alert-Tired	3.73 (1.08)	3.91 (0.70)	3.79 (1.08)
Calm-Agitated	4.03 (0.67)	4.18 (0.76)	4.23 (1.07)
KKL			
Selective Attention I: Numbers	4.58 (3.69)	4.67 (3.34)	5.08 (3.51)
Selective Attention I: Letters	4.33 (3.78)	4.17 (3.69)	5.50 (3.43)
Selective Attention II	69.32 (41.34)	75.64 (37.39)	73.81 (38.14)
Confrontation Naming	5.00 (2.59)	5.83 (2.32)	5.04 (2.99)
Short-Term Memory	3.13 (2.71)	3.54 (2.41)	3.42 (2.80)
Long-Term Memory	1.67 (2.24)	2.25 (1.89)	2.33 (2.62)
Coordination	2.58 (0.78)	2.83 (0.48)	2.63 (0.77)

**Table 3:** Paired *t*-test results for scores collected after one-week Jymmin’ intervention compared to scores collected after one week of passive listening condition.

Domain	Mean Score after Jymmin’	Mean Score after Control	T-Value
MDMQ			
Good-Bad	4.12	4.32	−1.15
Alert-Tired	3.94	3.80	0.58
Calm-Agitated	4.11	4.26	−0.49
KKL			
Selective Attention I: Numbers	4.96	4.79	0.31
Selective Attention I: Letters	4.71	4.96	−0.36
Selective Attention II	78.96	70.48	1.21
Confrontation Naming	5.79	5.08	1.71
Short-Term Memory	4.04	2.92	* 2.33
Long-Term Memory	2.33	2.25	0.19
Coordination	2.75	2.71	0.21
Length of Time	12.67	10.67	** 3.03

Note: * Significant at *p* < 0.05 level. ** Significant at *p* < 0.01 level.

## Data Availability

The first (J.V.S.) or corresponding (T.F.) authors can be contacted with requests for access to the data.
